# Quantitative analysis of adsorption and desorption of volatile organic compounds on reusable zeolite filters using gas chromatography

**DOI:** 10.1371/journal.pone.0227430

**Published:** 2020-01-08

**Authors:** Jeongjun Lee, Jihyun Jeon, Jaehyuk Im, Junhwan Jang, Jaegun Lee, Hee-Jung Choi, Beom-Rae Noh, Kyoung-Kook Kim, Soohaeng Cho

**Affiliations:** 1 Department of Physics, Yonsei University, Wonju, South Korea; 2 Department of Nano-Optical Engineering, Korea Polytechnic University, Siheung, South Korea; Institute of Materials Science, GERMANY

## Abstract

In this study, we propose a method to quantitatively analyze the concentration of VOCs adsorbed on zeolite filters via gas chromatography (GC). The sampled VOCs from the filters with ethanol as a solution were characterized using GC to determine the concentration of the adsorbed VOCs by comparing the areas of GC peaks of the detected VOCs and ethanol. The proposed method also enabled determination of the desorption (regeneration) conditions of the zeolite filters according to heating temperature and time for various VOCs. Repeated adsorption and desorption of VOCs on zeolite filters and GC analyses allow us to evaluate the durability and reusability of the filter and could help predict the lifetime of zeolite filters in practice.

## Introduction

As the manufacturing of semiconductors has increased over the years, interest in the air pollution and safety issues arising from the manufacturing processes is increasing[1, 2]. During these processes, various volatile organic compounds (VOCs) are generated, and many studies are in progress on their collection and removal[3, 4]. VOCs, such as toluene (C_6_H_5_CH_3_), o-xylene {C_6_H_4_ (CH_3_)_2_}, trichloroethane (TCA, C_2_H_3_Cl_3_), ethylene glycol monoethyl ether (EGM, C_4_H_10_O_2_), and trichloro-ethylene (TCl, C_2_HCl_3_), are common air pollutants emitted by chemical, petrochemical, and semiconductor industries[5]. Prolonged exposure to VOCs is fatal for the human body, and the major potential effects on health include acute and chronic respiratory effects, neurological toxicity, lung cancer, and eye and throat irritation[6]. Fatigue, nausea, sore throat, and watery eyes caused by indoor air quality (or ‘sick building’ syndrome) are often induced by VOCs, ozone, NO, and bacteria[7]. Various technologies have been investigated for decomposing and filtering VOCs, including catalytic oxidation, thermal decomposition, nano-carbon based VOC absorbers[8, 9], and condensation using inorganic filters[10], bio filters[11, 12], TiO_2_ nano-photocatalysts[13], plasma[14], and zeolite filters[15]. Among them, zeolite has been known to effectively adsorb VOCs [16] and the zeolite filter is employed in a commercial VOC reduction systems and can be regenerated and reusable by heating. However, research and development to test the durability and reusability of the filters are still required.

In this study, we propose a method to analyze the concentration of VOCs adsorbed by zeolite (ZSM-11) filters using GC. Various VOCs were adsorbed by the zeolite filters. The sampled VOCs with ethanol as the solution were characterized using GC to determine the concentration of the collected VOCs by comparing the areas of the GC peaks of the detected VOCs and those of ethanol. The desorption (regeneration) conditions of the zeolite filters according to heating temperature and time were determined for various VOCs. We performed repeated adsorption and desorption of VOCs on zeolite filters and GC analyses to evaluate the durability and reusability of the filter, thus providing a way to predict the lifetime of zeolite filters.

## Materials and methods

### Materials: Volatile organic compounds

VOCs are a large family of carbon-based chemicals with more than 300 types of members. The definition of VOCs varies based on the country and organization, but all VOCs have the general characters of a low boiling point, high vapor pressure, and strong reactivity, especially with respect to photochemical reactions. VOCs used in this study are toluene, propylene glycol methyl ether acetate (PGMEA), ethyl benzene and trichloroethylene. Each VOC has distinct characteristics such as boiling point, polarity, and molecular weight as shown in Table 1.

**Table 1:** Characteristics of VOCs.

VOCs	Formula	Molecular weight (g/mol)	Polarity	Boiling point (°C)	Concentration (%)	CAS NO.
**Ethanol**	C_2_H_5_OH	46.07	polar	78.24	Absolute	64-17-5
**Toluene**	C_6_H_5_CH_3_	92.14	nonpolar	111	99.5	108-88-3
**PGMEA**	C_6_H_12_O_3_	132.16	-	146	99.0	108-65-6
**Ethylbenzene**	C_8_H_10_	106.17	nonpolar	136	99.8	100-41-4
**Trichlorethylene**	C_2_HCl_3_	131.4	polar	87.2	99.0	79-01-6

### Sampling process (GC sampling)

**Fig 1:**
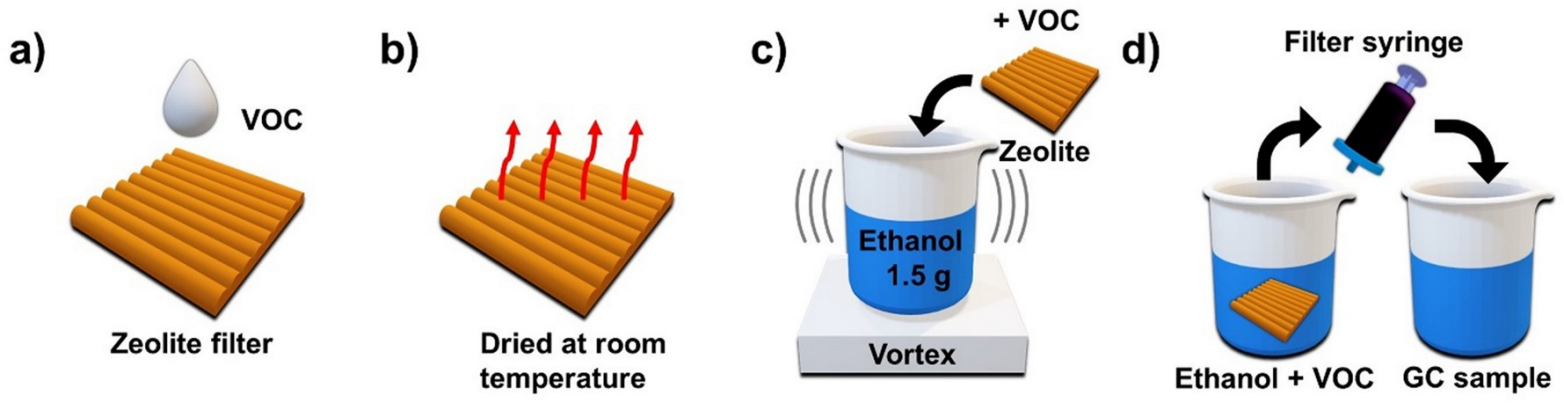
GC sampling process. (a) Adsorption of VOCs on the zeolite filter. (b) The filter was dried at room temperature (20 °C). (c) The filter was cut into 2 × 3 cm^2^ and immersed in 1.5 g of ethanol, followed by shaking. (d) The solution was extracted using a syringe and filtered to make a sample for GC measurement.

The sampling process for GC measurement is schematically described in Fig 1 VOCs were adsorbed on the zeolite filter and dried for 1 hour at ~20 °C (Fig 1a and 1b). The zeolite filters were cut into 2 × 3 cm^2^ pieces, placed in a beaker with 1.5 g of ethanol, and shaken on a vortex (Fig 1c). Samples of the VOC and ethanol solution were obtained with a syringe and filtered for GC measurement (Fig 1d).

### Super-saturated adsorption (Liquid adsorption)

Super-saturation adsorption was performed as follows. First, we placed a zeolite filter on a stainless-steel plate and sprayed the target VOCs on it to wet the filter thoroughly. The filter was dried at room temperature for 1 hour at 20 °C. The dried filter piece was separated by 2 × 3 cm^2^, and GC sampling was performed.

### Gas adsorption

The gas adsorption experiment was performed in the following way. The gas concentration of each VOC was 10 ppm. In the test chamber, VOCs were adsorbed on the filter, and GC sampling was performed on the 2 × 3 cm^2^ samples.

### Regeneration of zeolite filter using a furnace

A furnace (HTF-Q100, Hantech Corp.) was used to regenerate the filter by thermal desorption. To set the thermal regeneration conditions for each VOC, the following procedure was carried out. The supersaturated adsorption filter was heated using a furnace at various temperatures and heating times. The regenerated (thermally desorbed) filter was sampled for GC analysis using a sample size of 2 × 3 cm^2^. GC measurements of each sample allowed to determine the optimized filter heating conditions for each VOC by repeatedly comparing the residual amounts of VOCs versus supersaturated adsorption: toluene: 200 °C in furnace for 60 minutes, PGMEA: 150 °C in furnace for 30 minutes, ethylbenzene: 80 °C in furnace for 30 minutes, and trichloroethylene: 60 °C in furnace for 5 minutes.

### GC analysis

The prepared samples were analyzed by GC (GC; YL6100 Gas Chromatography, Youngin Chromass Co.). The columns used in the experiments were DB-WAX UI (Column; Agilent Technologies), and the carrier gas was N_2_. The detector type was a flame ionization detector (FID). The temperatures of the inlet and detector were 150 °C and 300 °C, respectively, and the oven temperature was set at 100 °C. Measurements were obtained in isothermal mode.

**Fig 2:**
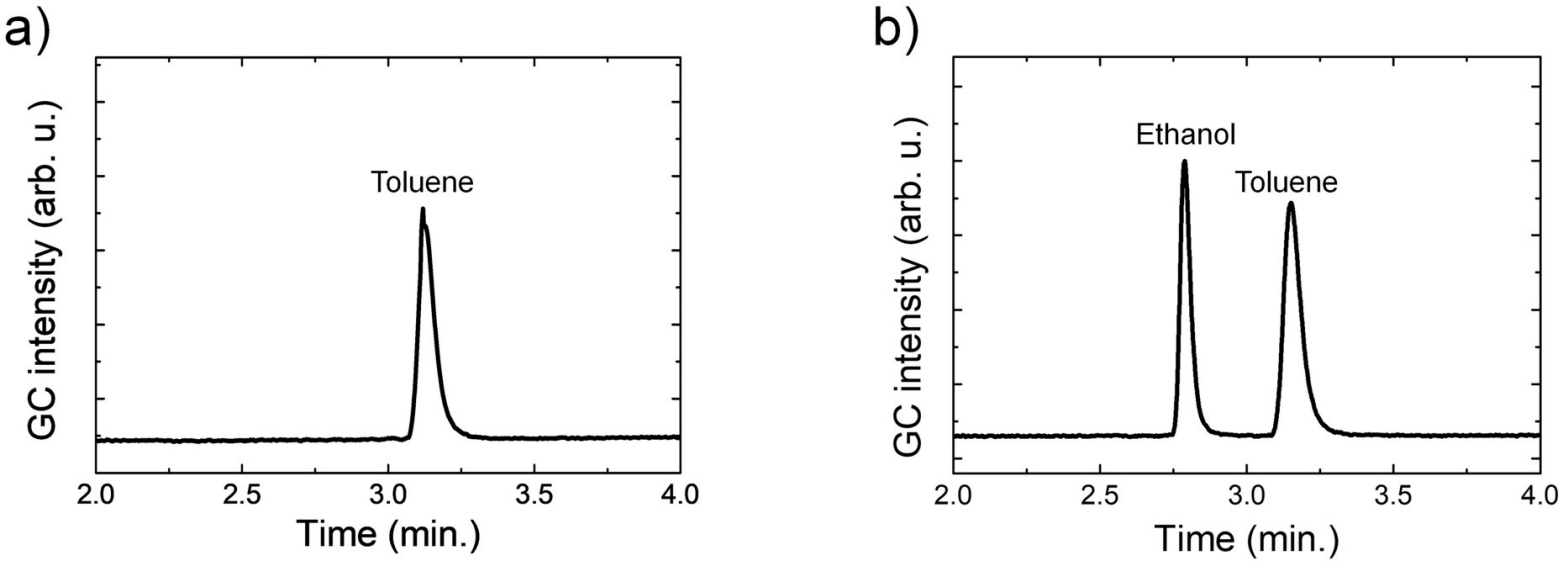
GC analysis data. (a) Toluene peak time, and (b) toluene and ethanol solution peak times.

Fig 2a shows the GC peak from toluene, and Fig 2b shows the GC peaks of the mixed solution of toluene and ethanol. We confirmed that GC peak time was constant and distinguishable for each VOC and ethanol.

### Standard calibration

The ratio of the GC peak areas for each substance was measured differently from the mass ratio of the actual VOC mixed solution depending on the type of column used for GC measurements and the detector. To determine the mass of the actual VOC contained in the zeolite filter, the correction value was multiplied, so that the peak area ratio of the VOC material obtained from the GC measurement matched the mass ratio of the actual VOC material. The equation is as below:

$$m_{VOC}=(V_{VOC}+V_{ethanol})[\frac{1}{\left(\frac{\alpha }{D_{ethanol}})+(\frac{1}{D_{VOC}}\right)}]$$ (1)

where *m* is mass, *V* is volume, *D* is density, and *α* is the ethanol peak area ratio obtained by GC measurement multiplied by the correction factor *α*. The peak area ratio should be 1:1 when a solution of ethanol and VOC in a mass ratio of 1:1 is analyzed by GC. The GC peak area ratio data obtained from the repeated measurement were multiplied by the correction factor to make the mass ratio 1:1. The correction factors for each VOC are listed in Table 2.

**Table 2:** Correction factors for each VOCs.

VOCs	Toluene	PGMEA	Trichloroethylene	Ethylbenzene
**Correction factors**	2.08	0.85	1.06	1.99

In the above equation, α was obtained by multiplying the ethanol peak area ratio obtained by GC measurement with the correction value. Using the GC peak area data of the VOC material compared with that of ethanol obtained from the experiment, the content of the actual VOC material was calculated by the above formula. Because the mass of ethanol used in the GC sampling process is known, the mass of VOCs adsorbed on the sampled adsorbent can be determined. The content of VOCs can be analyzed according to the peak area based on the data obtained by making a standard calibration solution and via GC measurement.

**Fig 3:**
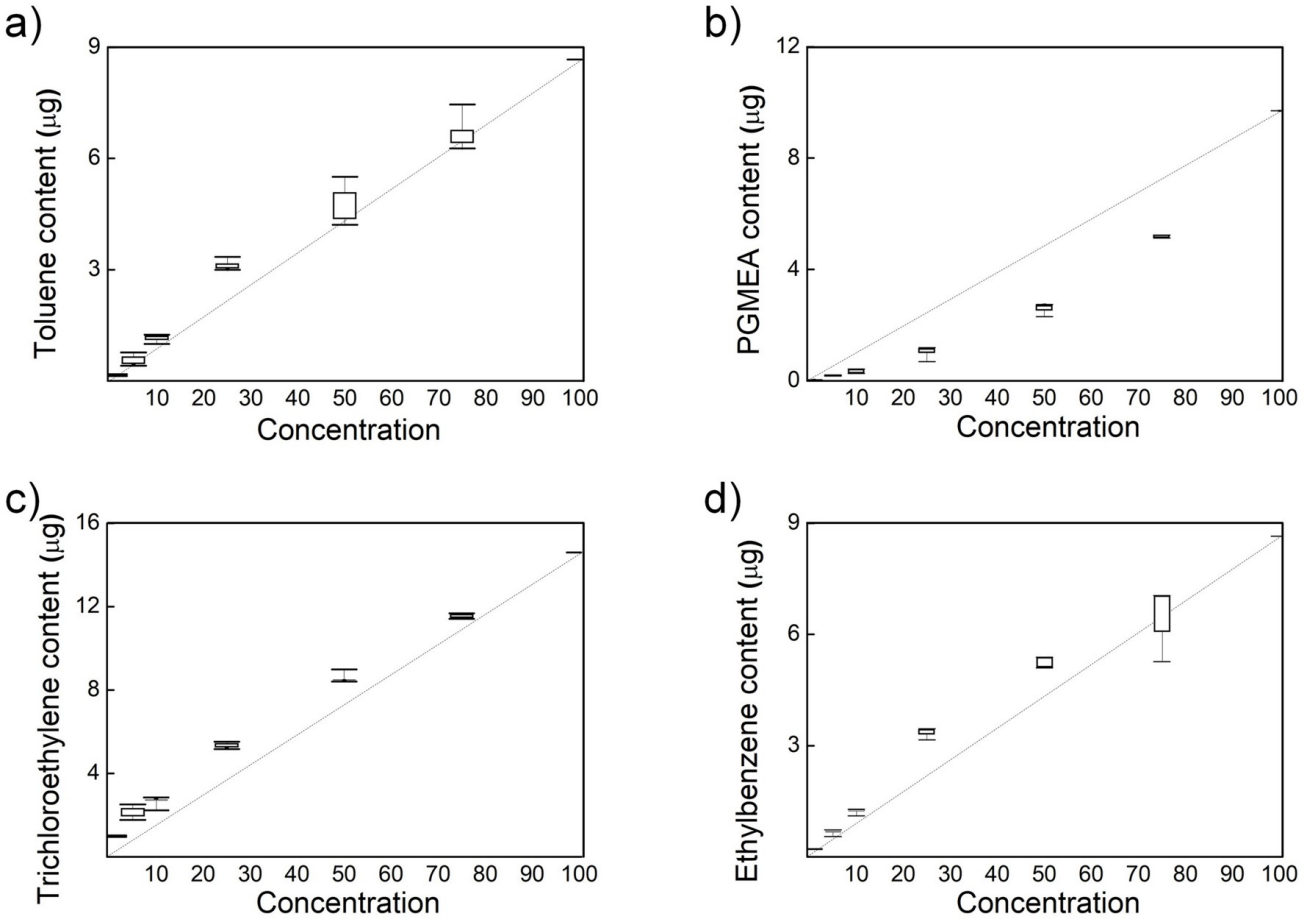
VOC content data obtained using a standard calibration solution of concentration of VOC. (a) Toluene, (b) PGMEA, (c) Trichloroethylene, and (d) Ethylbenzene.

The VOCs (toluene, PGMEA, ethylbenzene, and trichloroethylene) and ethanol were mixed to prepare 1, 5, 10, 25, 50, 75 and 100 wt% VOC standard calibration solutions, which were analyzed by GC as shown in Fig 3. The dotted lines in Fig 3 are the ideal not the linearly fitted values to demonstrate that experimental and the ideal values have similar trends. The VOC content is estimated by comparing GC peak area ratio and wt%. As the concentration of the calibration solution increases, the amount of VOCs proportionally increases. Such linear relations confirm that GC measurements can be used to properly determine the content of VOCs in the GC sampling solution. The solution for each concentration was measured 5 times.

## Results and discussion

**Fig 4:**
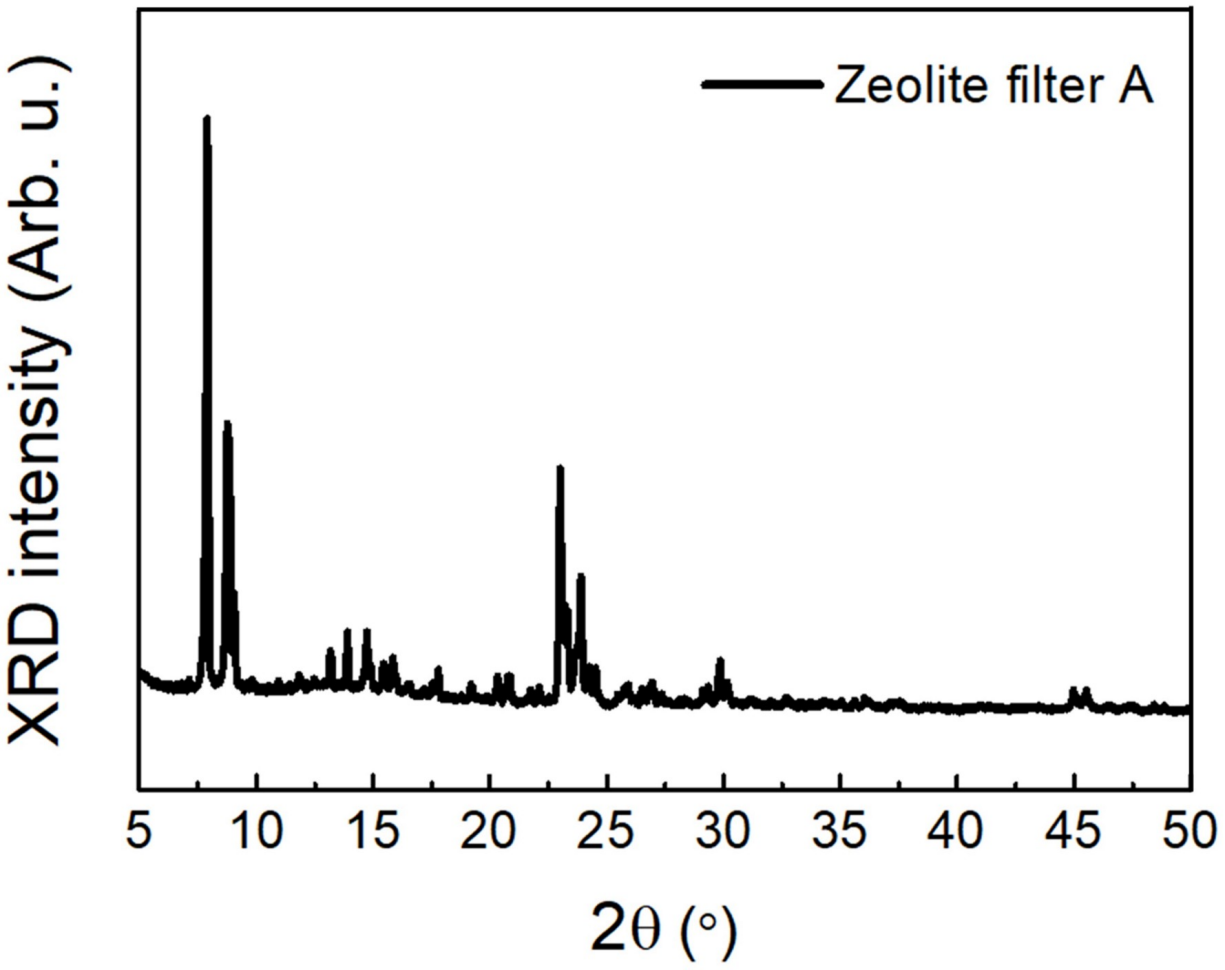
XRD pattern of zeolite filter A.

We prepared two types of zeolite filters for this study. One (zeolite filter A) is an industrial grade zeolite filter for VOC reduction for use in factories and high-volume clean rooms and is not cheap. The second filter (zeolite filter B) is a commercial and inexpensive zeolite filter that can be bought online and offline for home appliances such as a cat litter box for comparison with the industrial filter. X-ray diffraction (XRD) measurement was performed to confirm the structure type of the zeolite filter A, which is the main subject of this work, as shown in Fig 4. XRD analysis was carried out on a Bruker-D2 Phaser X-ray diffractometer equipped with Cu Ka radiation and operated at 40 mA and 40 kV. The comparison of the measured peaks with the reported XRD peaks for various types of zeolites[17] indicates that the type of the zeolite filter A is ZSM-11.

**Fig 5:**
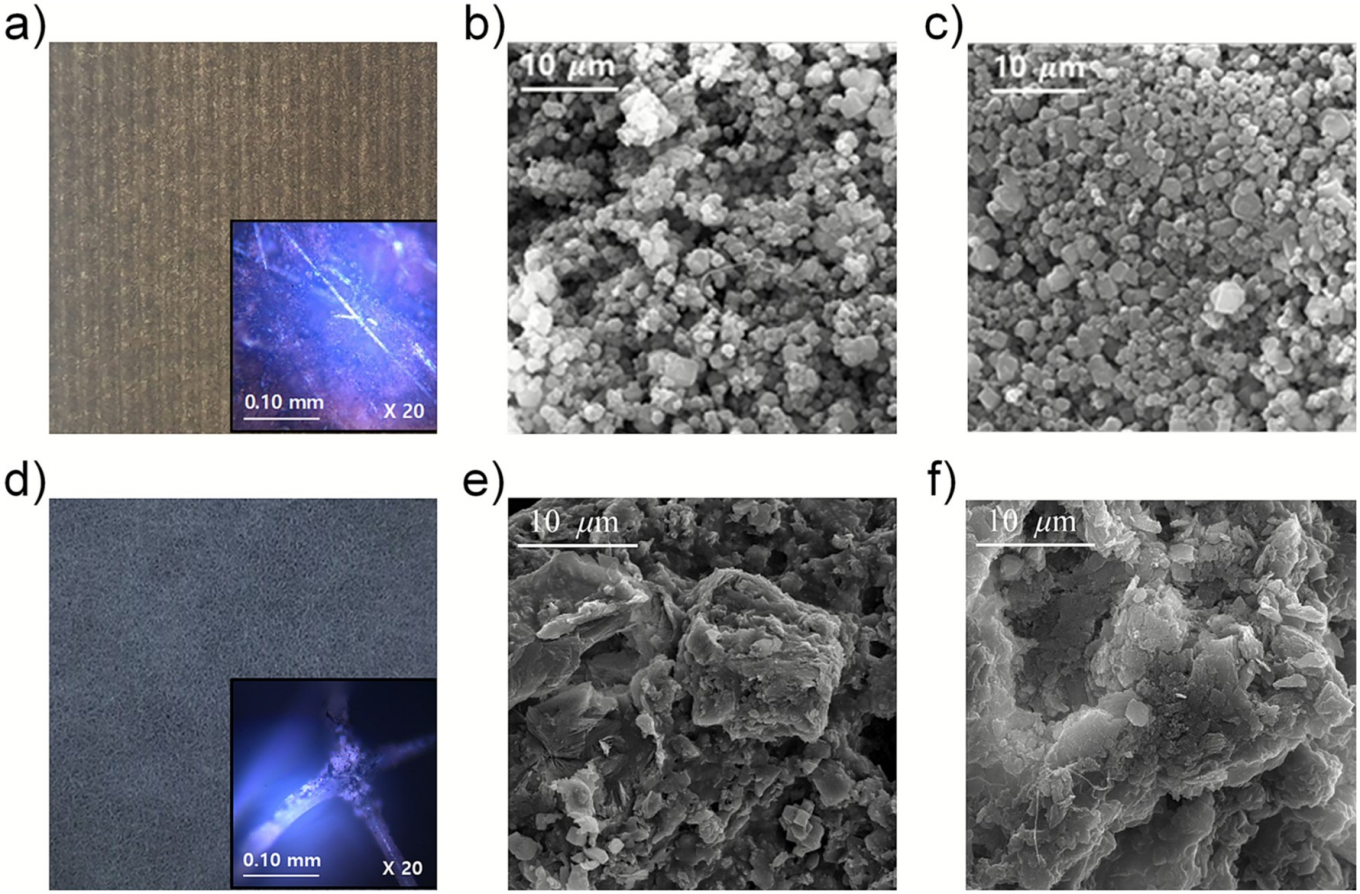
Comparison of SEM and microscopy images for adsorbed and desorbed zeolite filters. (a) Photograph and optical microscopy image (inset, ×20 magnification) of zeolite filter A. (b) SEM image of zeolite filter A with 10 ppm toluene gas adsorbed for 60 minutes. (c) Zeolite filter A regenerated at 200 °C for 60 minutes. (d) Photograph and optical microscopy image (inset, ×20 magnification) of zeolite filter B. (e) SEM image of zeolite filter B saturated with toluene. (f) Regenerated zeolite filter B at 200 °C and 60 minutes.

In Fig 5a and 5d, the optical microscopy image shows the presence of zeolite crystals and amide fibers on the filter. The zeolite filter A (TOYOBO Co., Japan) shown in Fig 5a includes a corrugated cardboard adhered to paper. The filters with adsorbed and desorbed with toluene were analyzed using scanning microscope (SEM; Quanta 250FEG, Thermo Fisher scientific Co.) as shown in Fig 5b, 5c, 5e and 5f. EDS (EDS; Apollo X, AMETEK Materials Analysis Division Co.) was also utilized. Spherical particles can be observed in Fig 5b and 5c. The diameter of zeolite was found to be between 0.9 μm and 2.2 μm. Zeolite filter B (Vanness Co., USA) is a sponge-like filter with zeolite particles attached to the fibers, as shown in Fig 5d. The zeolite grains in this filter are larger than those in filter A. However, it was found that the toluene adsorption and desorption states could not be clearly distinguished through SEM images of both filters.

Then, we performed EDS measurements to test if we can detect the contents of the adsorbed toluene on the filter. Table 3 shows the results of the EDS analysis of the zeolite filter A when toluene adsorption and regeneration cycles were repeated for 10 and 20 times. The atomic components of VOCs adsorbed on the zeolite filter can be analyzed by EDS[18]. We predicted that the carbon content will vary with the amount of VOCs adsorbed on the zeolite filter. Because it was difficult to detect hydrogen in EDS, the amount of toluene adsorbed on the zeolite was measured by comparing the content of carbon[19]. EDS analysis showed that the carbon ratio of the toluene-adsorbed filter was higher than that of the filter exposed to air. It was also confirmed that the carbon ratio of the zeolite filter increased after repeated cycles of regeneration. When the filter is heated and regenerated at high temperatures, the VOCs penetrate into the filter, which makes it difficult to regenerate the filter[20]. This means that the durability of the filter is limited. However, analyzing the durability of the filter as the number of cycles increases is difficult using EDS because there was no consistent tendency in change of the carbon contents of the adsorbed and regenerated filters. Furthermore, it is also impossible to distinguish which VOC is adsorbed on the filter. These results indicate that SEM and EDS are inappropriate for qualitative analysis of VOCs.

**Table 3:** Atomic percentage of carbon, oxygen, and silicon after repeated cycles of supersaturated adsorption of toluene on the zeolite filter A and regeneration of the filter for 10 and 20 times.

		C	O	Si	*Sum^*a*^
**(a) Zeolite filter exposed in air**		17.3	46.15	36.54	99.99
**(b) 10 cycles**	**Adsorption**	20.25	50.03	28.32	100
	**Regeneration**	0	59.28	40.72	100
**(c) 20 cycles**	**Adsorption**	21.42	49.06	28.56	100
	**Regeneration**	12.72	57.71	29.56	100

*Sum^*a*^: The zeolite filter is coated with Pt, so EDS analysis contains Pt as an extraction element.

**Fig 6:**
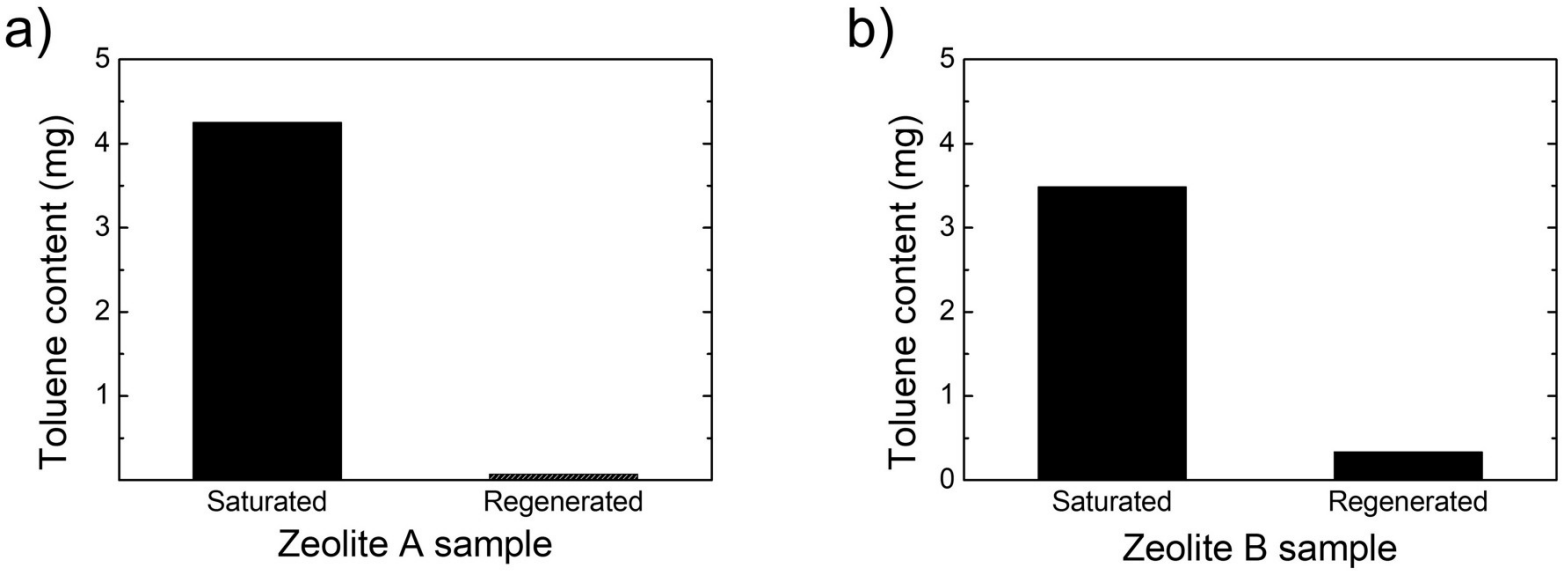
Comparison of toluene contents in the saturated and regenerated samples. (a) Zeolite filter A. (b) Zeolite filter B.

Fig 6 Shows a comparison of the contents of toluene analyzed by GC in the supersaturated adsorption filters and the regenerated filters in the furnace at 200 °C for 60 minutes. It was confirmed that VOC adsorption and regeneration filters can be clearly distinguished by GC analysis. Although the toluene-adsorbed zeolite filter was regenerated at high enough temperature and for long enough time, the toluene remained in zeolite filter. This is because the adsorbed substance diffuses deeper into the zeolite crystal structure by applying heat. After desorption of zeolite A and B, the amount of remaining toluene is different. It is explained that the degree of internal diffusion differs according to the zeolite crystal structure. In the following experiments which will be shown later, the degree of desorption of VOC was confirmed by heating temperature and time, and it was judged that the zeolite filter was regenerated if remaining VOC content is 10% less than that for the supersaturated filter. From the above results, it was confirmed that qualitative and quantitative analysis of VOC can be performed by GC analysis of various zeolite filters. All subsequent experiments were carried out using the zeolite filter A only.

**Fig 7:**
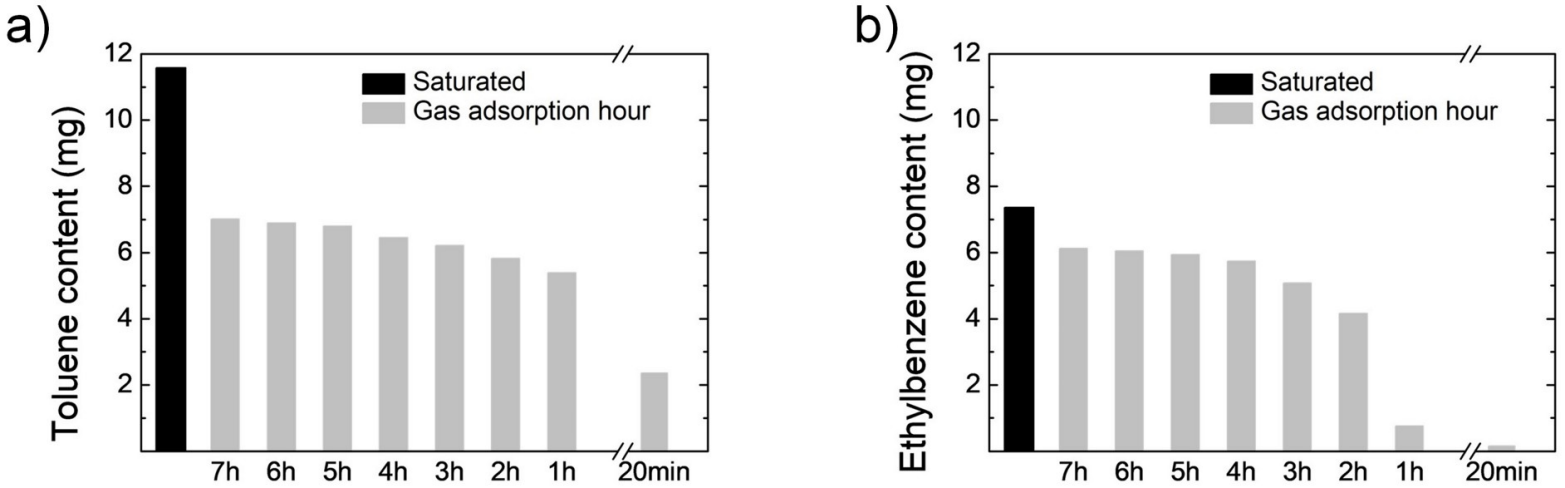
VOC contents with adsorption time. (a) Toluene gas, (b) Ethylbenzene gas.

VOC contents as a function of the adsorption time were investigated. In this case, two target VOCs (toluene and ethylbenzene) were adsorbed on the zeolite filter using nitrogen bubbling setup in the test chamber. The concentration of each VOC gas was 10 ppm. The amounts of gas-adsorbed VOCs on the filter from 20 minutes to 8 hours were compared to each other as well as to the supersaturated adsorbed sample, as shown in Fig 7. As expected, although not linear, the amount of adsorbed VOC on the zeolite filter increased with adsorption time. In addition, the saturation of adsorption was clearly confirmed when the adsorption time is longer than 6 hours. For comparison, the supersaturated adsorption value is also displayed.

**Fig 8:**
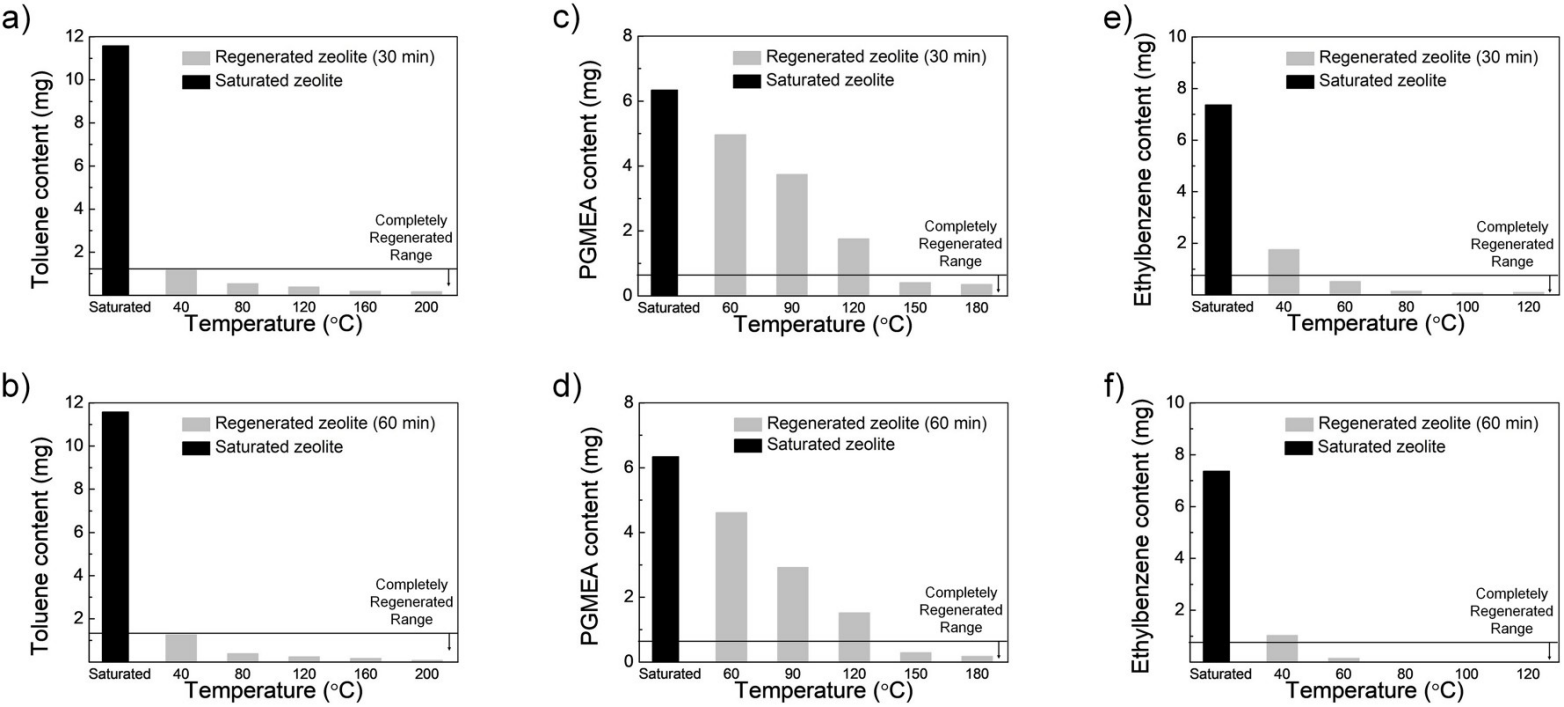
VOC contents as per heating temperature. (a) Toluene 30 minutes, (b) toluene 60 minutes, (c) PGMEA 30 minutes, (d) PGMEA 60 minutes, (e) ethylbenzene 30 minutes, and (f) ethylbenzene 60 minutes.

To obtain the optimal conditions for thermal regeneration of zeolite filters (desorption process), the saturated zeolite filters with VOCs (toluene, PGMEA, and ethylbenzene) were heated using a furnace. Fig 8a, 8b and 8e show the residual VOC contents after heating at various temperatures for 30 minutes, and Fig 8b, 8d and 8f show the data for 60 minutes. It was confirmed that the amount of residual VOC contents decreases with increasing regeneration temperature and time. When the residual VOC contents are less than 10% of that for the supersaturated filters, we consider that the filters were fully regenerated because neither the longer heating process time nor higher heating temperature further reduced the residual contents. Based on these results, we determined that the proper regeneration conditions for the zeolite filter A according to VOCs are 60 minutes at 200 °C for toluene, 30 minutes at 150 °C for PGMEA, 30 minutes at 80 °C for ethylbenzene, and 5 minutes at 60 °C for trichloroethylene, respectively.

**Fig 9:**
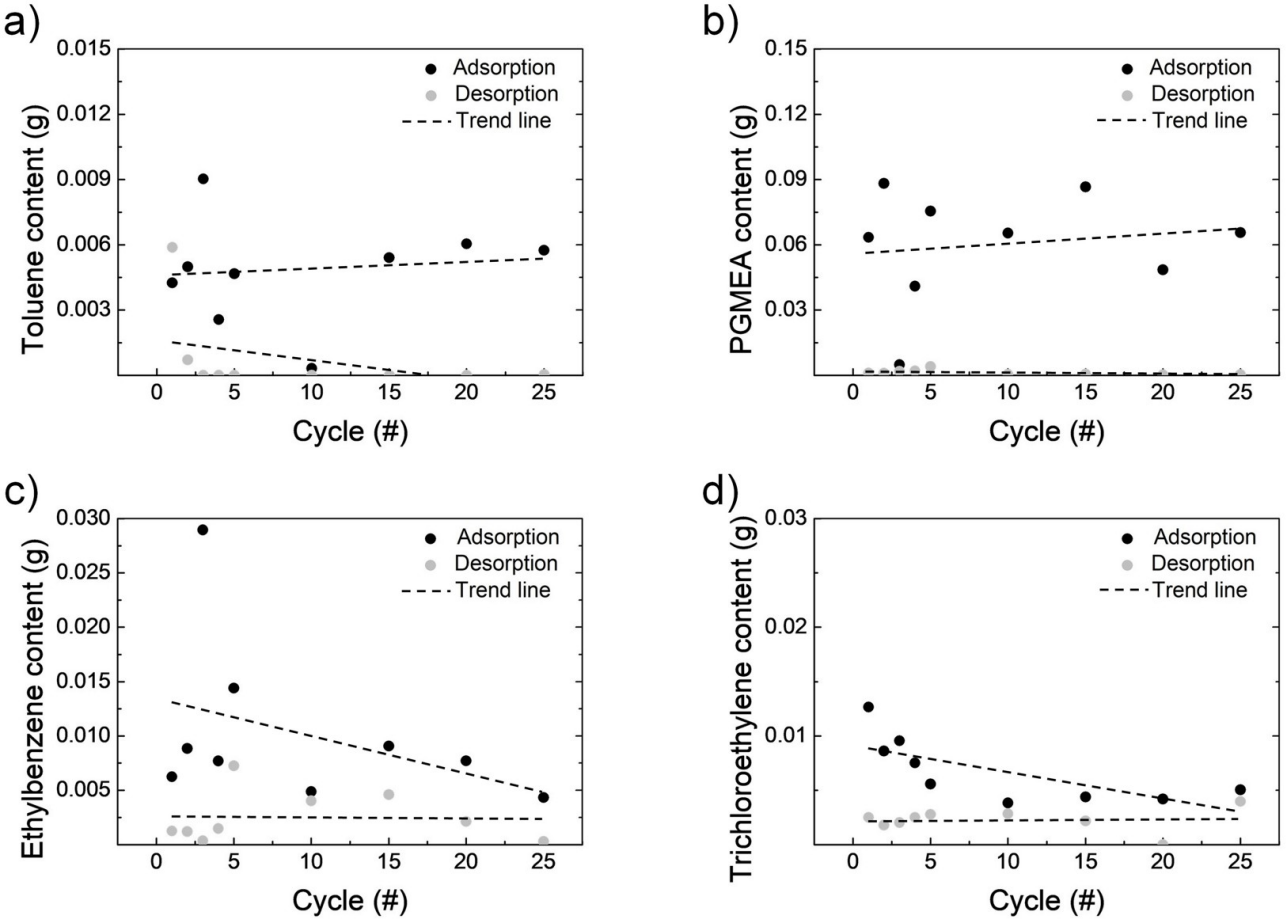
VOC contents based on the number of VOC adsorption and regeneration cycles. (a) Toluene, (b) PGMEA, (c) ethylbenzene, and (d) trichloroethylene.

Finally, to analyze the regeneration life of the zeolite filter, the VOC contents of the supersaturated and fully regenerated zeolite filters were measured repetitively. One cycle includes supersaturation and full regeneration processes. The regeneration conditions for each VOC were as explained previously. For each VOC (toluene, PGMEA, ethylbenzene, and trichlorethylene), 25 cycles were performed, and the amount of VOCs was analyzed by GC as shown in Fig 9. The adsorption tendency of VOCs in the zeolite filter according to the repetitive cycles was observed to vary depending on VOCs. The amount of adsorption of toluene was maintained without significant changes through repeated cycles (Fig 9a). In the case of PGMEA, the amount of adsorption increased with increasing number of cycles (Fig 9b). The adsorbed contents of ethylbenzene and trichlorethylene decreased with increasing number of cycles (Fig 9c and 9d). It can be seen that depending on the type of VOCs, the adsorption ability of the zeolite filter is maintained or decreased during repetitive regeneration. Based on these results, it is possible, especially when the target VOC and the zeolite filter type are decided, to predict the lifetime of the zeolite filters by repetitive measurements of VOC contents in the repeatedly adsorbed and regenerated zeolite filters.

## Conclusions

In this study, we proposed a method to analyze the concentration of VOCs adsorbed and desorbed on zeolite filters using GC, which was not possible with the conventional SEM and EDS measurements. The various VOCs (toluene, PGMEA, ethylbenzene, and trichlorethylene) were adsorbed by the zeolite filters (ZSM-11), and the sampled VOCs with ethanol as the solution from the adsorbed filters were characterized using GC to quantitatively determine the concentration of the collected VOCs by comparing the areas of GC peaks of the detected VOCs and those of the peaks of ethanol. We confirmed that the VOC contents of the adsorbed and regenerated zeolite filters can be clearly distinguished quantitatively by GC analysis. By means of the proposed method, the regeneration (desorption) conditions of the zeolite filters according to heating temperatures (60–200 °C) and times (30–60 minutes) were determined for various VOCs. Repeated adsorption and desorption of VOCs on the zeolite filters (25 cycles) and GC analyses allowed us to evaluate the durability and reusability of the filter and predict the lifetime of zeolite filters.
